# Supplementary figures and images for: High abundance of Early Miocene sea cows from Qatar shows repeated evolution of seagrass ecosystem engineers in Eastern Tethys
Source: PeerJ. 2025 Dec 10;13:e20030. doi: 10.7717/peerj.20030 (PMC12701702; doi:10.7717/peerj.20030)

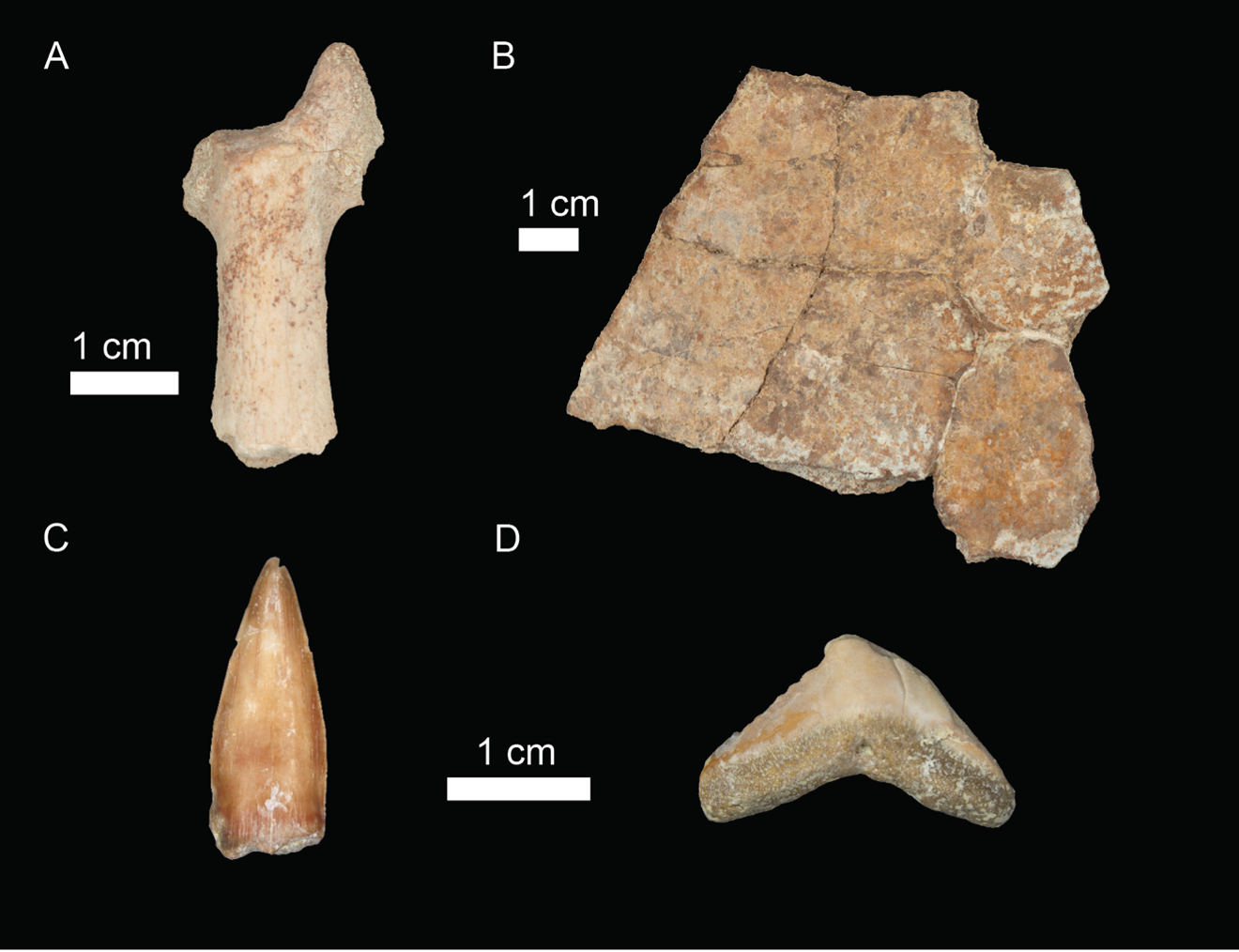

Supplement: Supplemental Information 1 — A, Ulna belonging to Odontoceti (ARC.2024.28.022). B, Carapace fragment from Testudines (ARC.2023.28.016). C, Tooth belonging to Sphyraenidae (ARC.2023.28.015). D, Tooth belonging to cf. Carcharhiniformes tooth (ARC.2023.28.012). [file peerj-13-20030-s001.png]

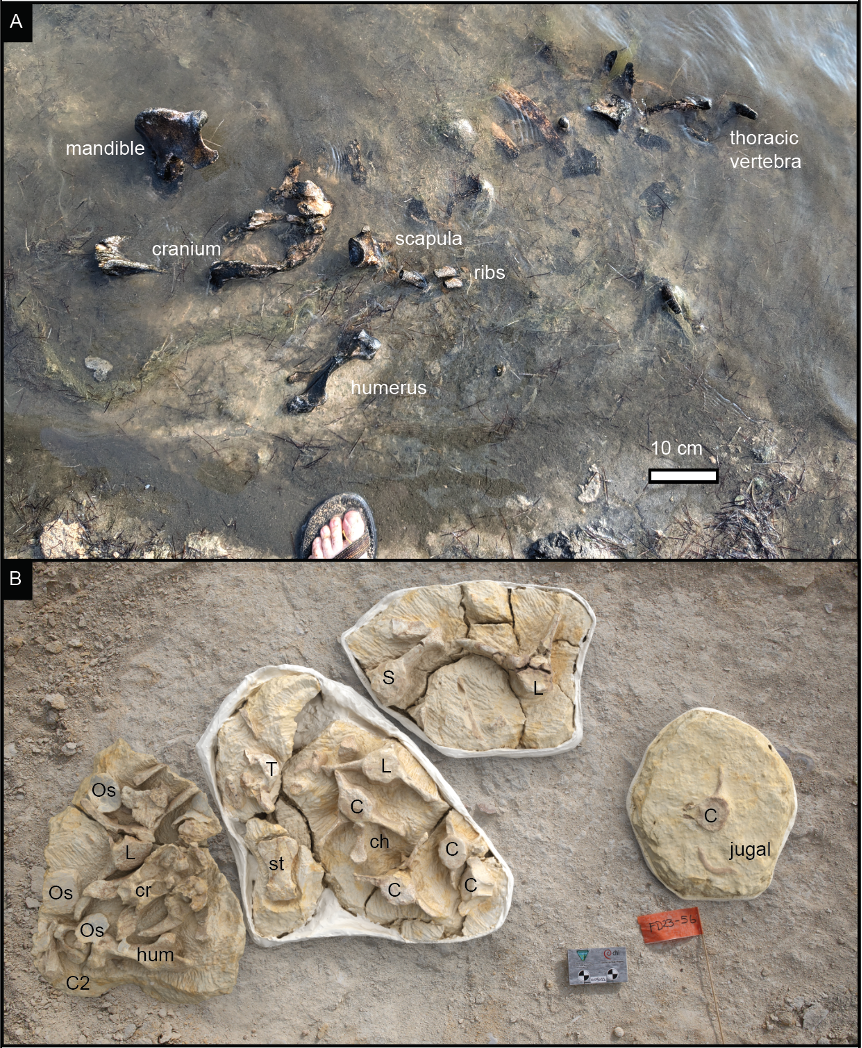

Supplement: Supplemental Information 2 — A, Dugong bones from the Hawar Islands, Kingdom of Bahrain, preserved in a mudflat. B, The type skeleton of Salwasiren with field jackets from locality FD 23-56 3D surface scanned prior to preparation but oriented according to their discovery in the field. Abbreviations: C, caudal vertebra; C2, axis, second cervical vertebra; ch, chevron; cr, cranium; hum, humerus; L, lumbar vertebra; Os, fossil oyster; S, sacral vertebra; st, sternum; T, thoracic vertebra. Scale bar = 5 cm. Image in (A) collected with the permission of the Supreme Council for the Environment and the Prime Minister’s Office for the Kingdom of Bahrain. [file peerj-13-20030-s002.png]

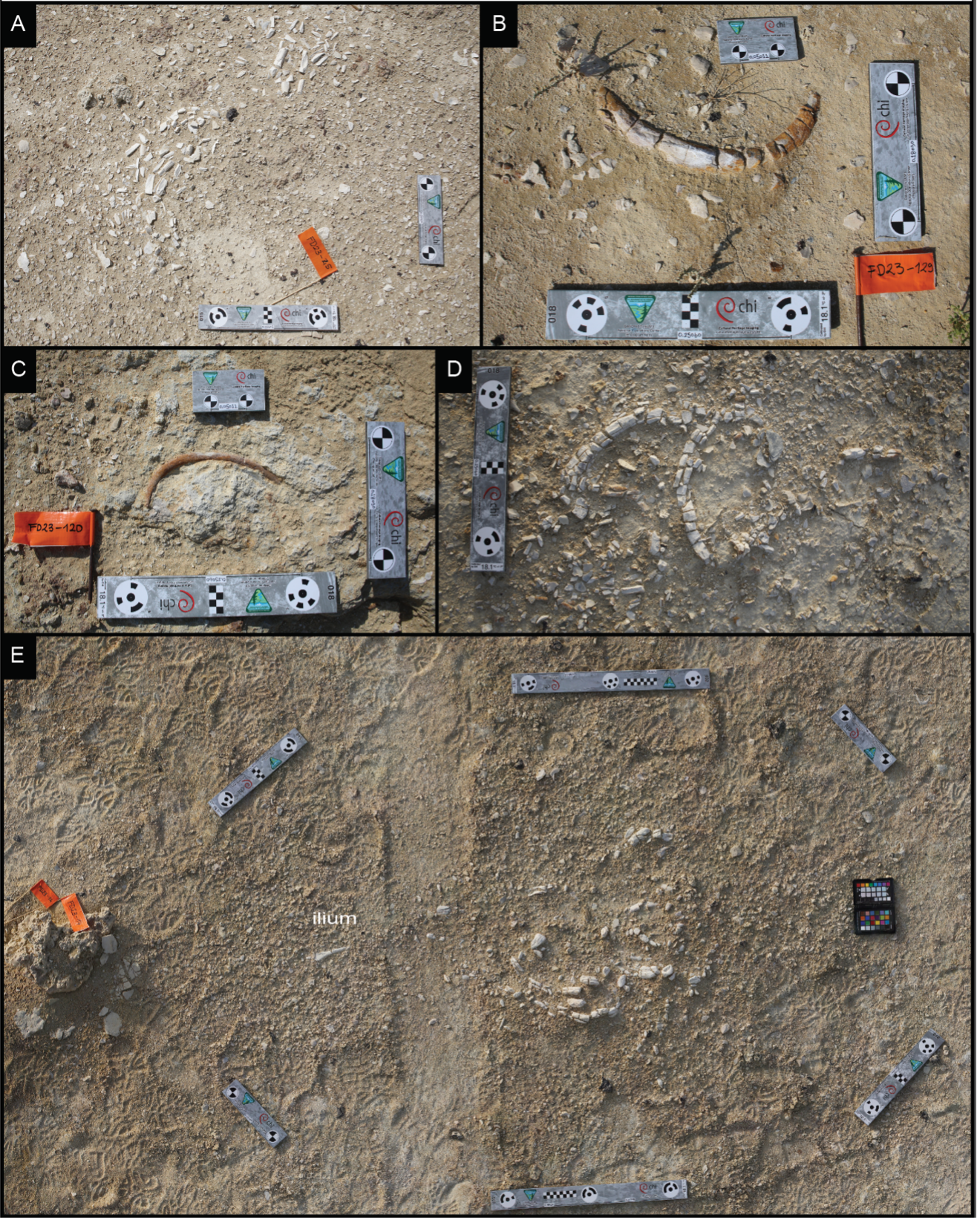

Supplement: Supplemental Information 3 — A-D, Ribs from localities FD 23-45, FD 23-129, FD 23-120, and FD 23-92 showing different preservation modes and E, a calibrated orthographic view of locality FD 23-14 generated by photogrammetry featuring disarticulated but associated set of ribs that likely belonged to a single individual fossil dugongid (representing Stage 2 of skeletal articulation, see Table S3). These fossils remain in situ, except for the ilium in (E), cataloged as ARC.2023.28.003. [file peerj-13-20030-s003.png]

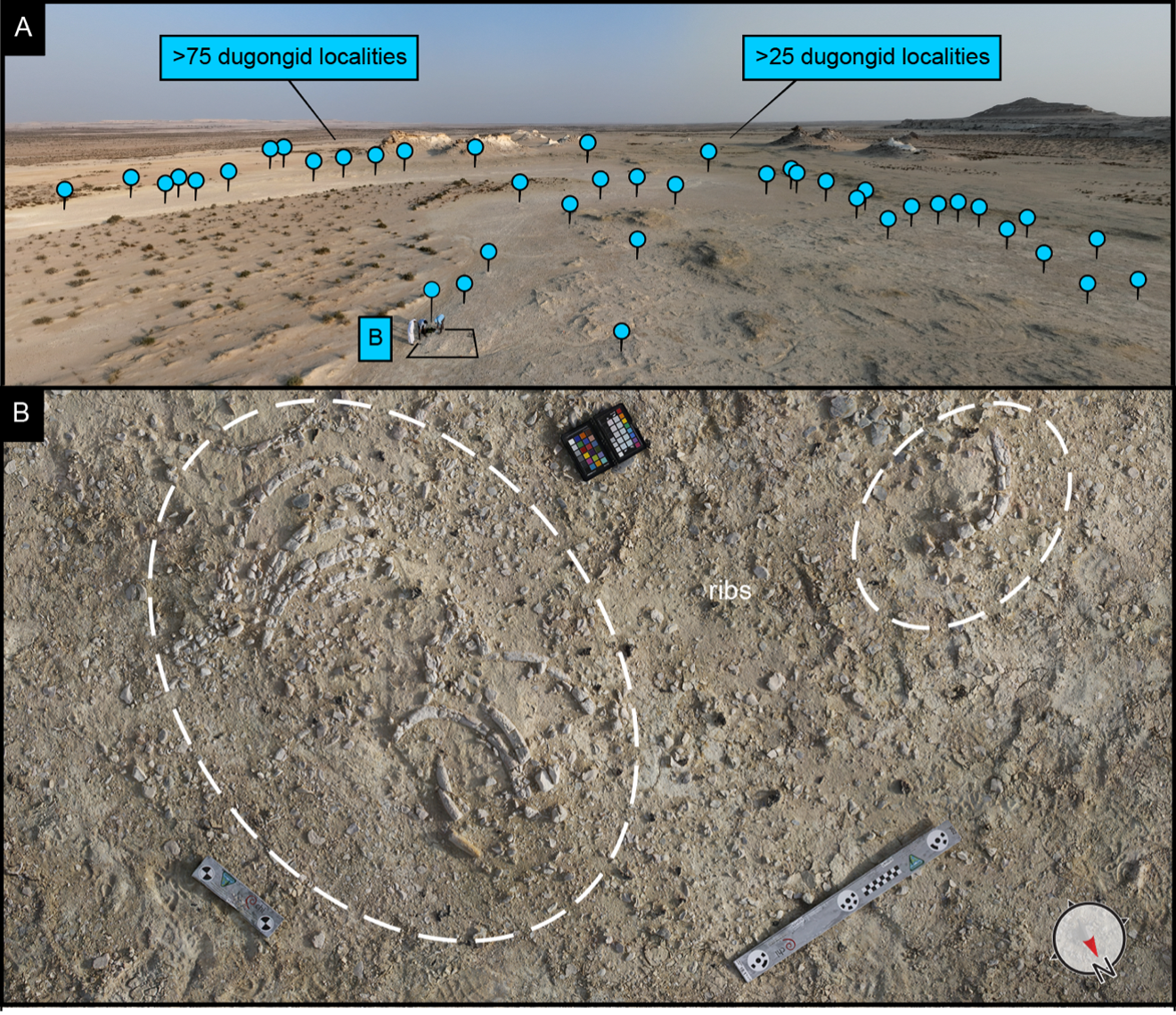

Supplement: Supplemental Information 4 — A, a view from the north near locality FD 23-75 and B, a calibrated orthographic view of locality FD 23-75 generated by photogrammetry featuring a disarticulated but associated set of ribs that likely belonged to a single individual fossil dugongid (representing Stage 2 of skeletal articulation, see Table S3). These fossils remain in situ. [file peerj-13-20030-s004.png]

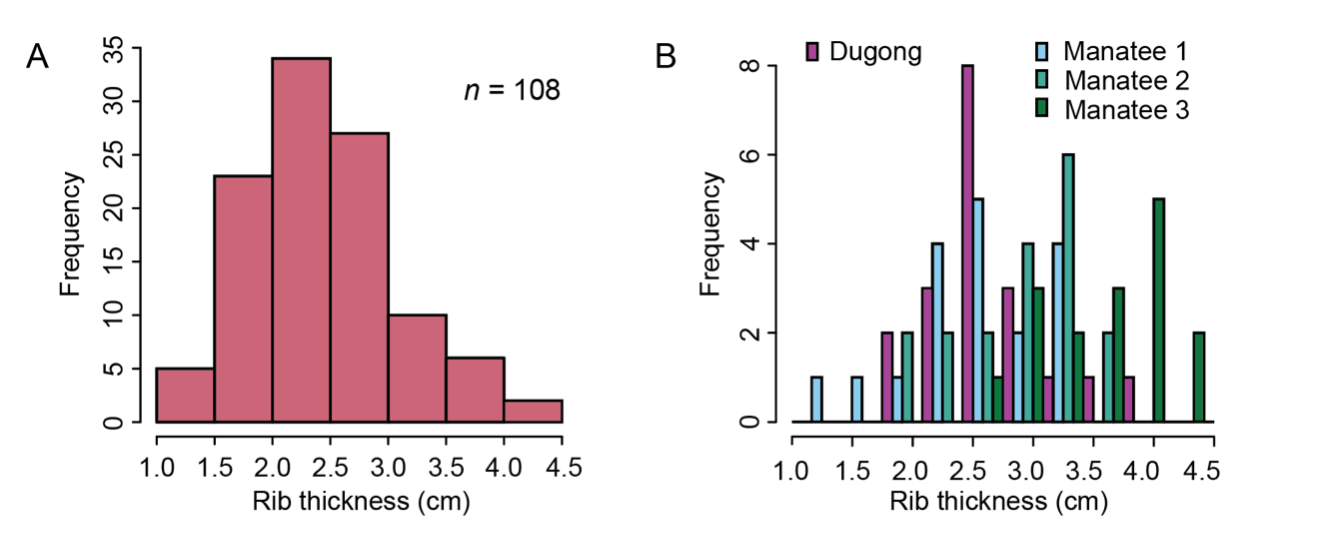

Supplement: Supplemental Information 5 — A, Distribution of maximum rib thickness for 108 fossil dugongids. B, Distribution for similar measurements in extant dugong and manatees. See Tables S5 for details. [file peerj-13-20030-s005.png]

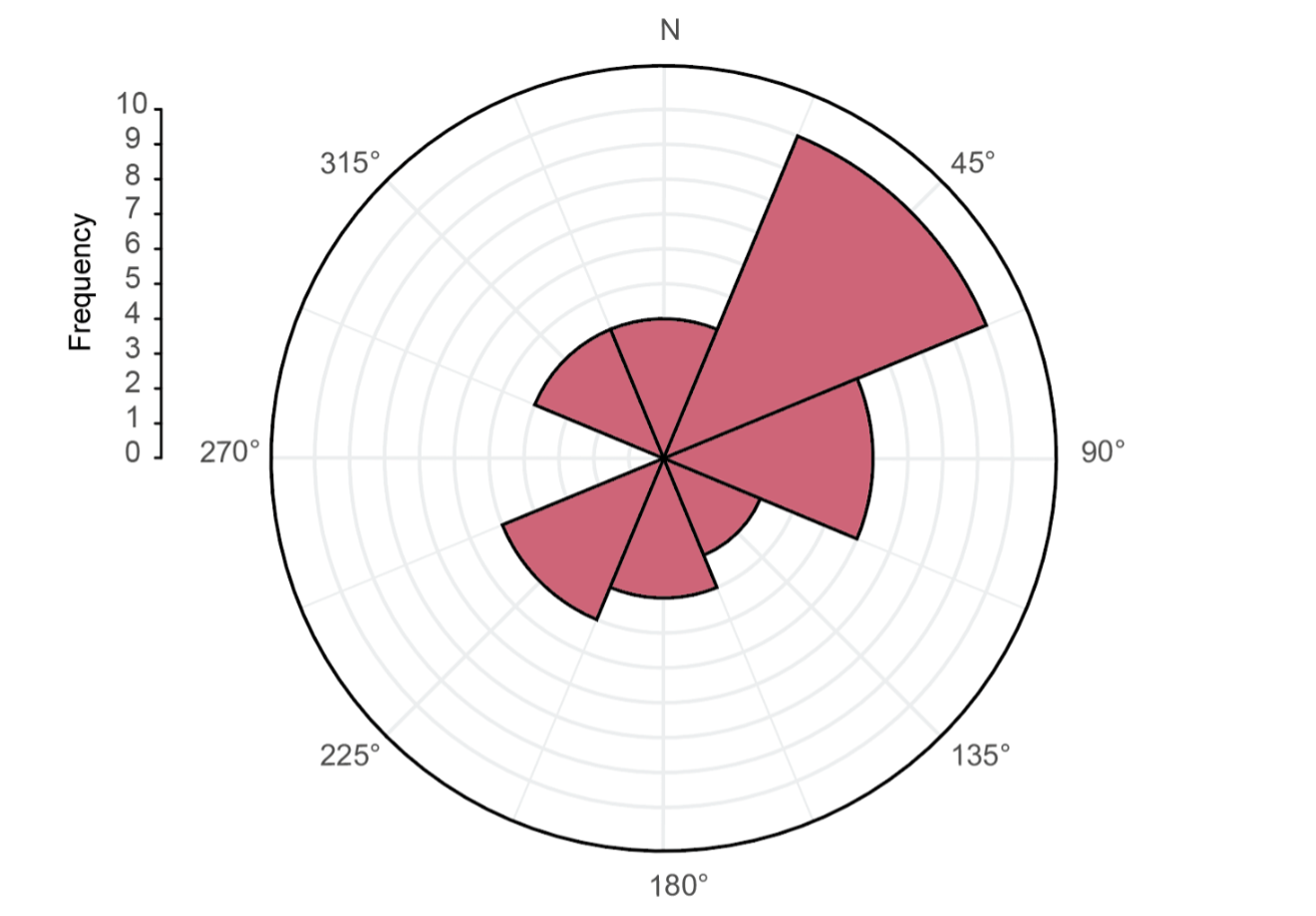

Supplement: Supplemental Information 6 — Rose diagram showing the distribution of major skeletal elements from fossil dugongids in three localities (i.e., FD 23-14, FD 23-56, and FD 23-75), relative to magnetic north. [file peerj-13-20030-s006.png]

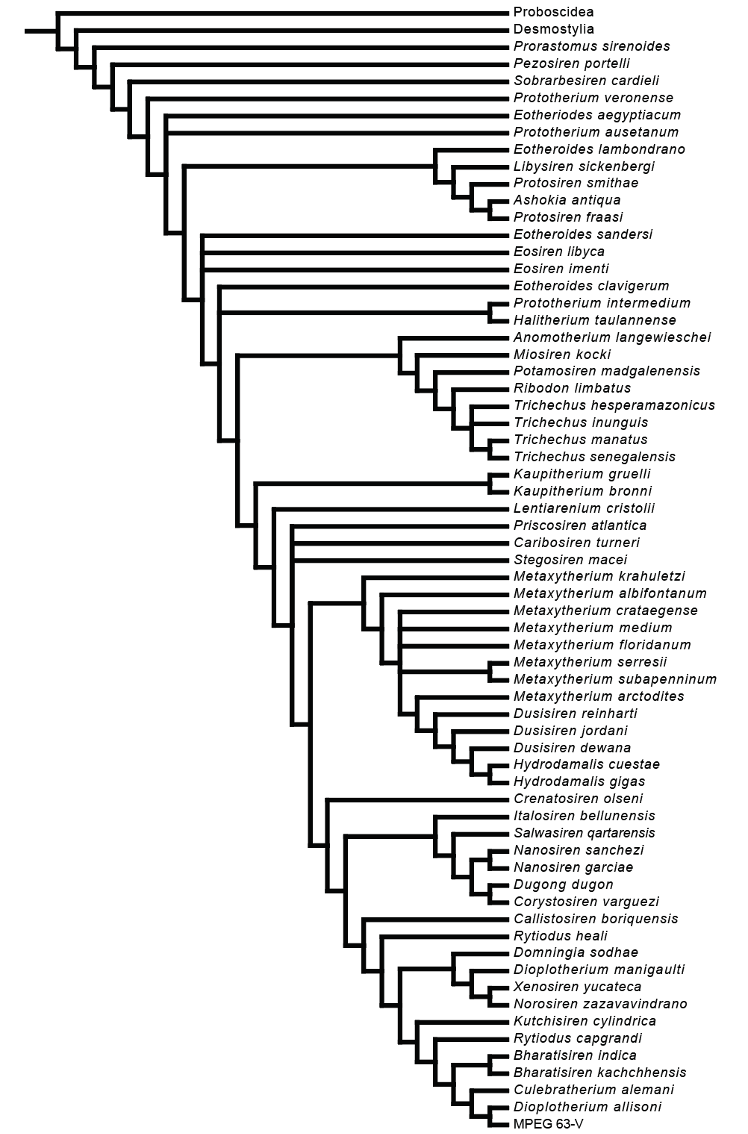

Supplement: Supplemental Information 7 — (180 MPTs of fit = 14.41, k = 9) from phylogenetic our analysis of Salwasiren qatarensis. Abbreviation: MPEG, Museu Paraense Emílio Goeldi, Belém, Pará, Brazil. [file peerj-13-20030-s007.png]
