# Supplementary material for: High abundance of Early Miocene sea cows from Qatar shows repeated evolution of seagrass ecosystem engineers in Eastern Tethys
Source: PeerJ. 2025 Dec 10;13:e20030. doi: 10.7717/peerj.20030 (PMC12701702; doi:10.7717/peerj.20030)
Supplement: Supplemental Information 8 [file peerj-13-20030-s008.docx]

Table S1. Skeletal element groupings based on fossil Dugongidae surveys at Al Maszhabiya, tabulated from 304 total minimum number of elements for fossil Dugongidae.

| **Skeletal element groupings** | **Count** |
| --- | --- |
| **All cranial elements (including FD 23-56)** | 11 |
| **All postcranial elements (including FD 23-56)** | 282 |
| **Any rib or rib combinations (minus FD 23-56)** | 252 |
| **% cranial elements of total MNE** | 3.62 |
| **% postcranial elements of total MNE** | 92.76 |
| **% ribs of total MNE** | 83.55 |
