# Supplementary material for: High abundance of Early Miocene sea cows from Qatar shows repeated evolution of seagrass ecosystem engineers in Eastern Tethys
Source: PeerJ. 2025 Dec 10;13:e20030. doi: 10.7717/peerj.20030 (PMC12701702; doi:10.7717/peerj.20030)
Supplement: Supplemental Information 9 [file peerj-13-20030-s009.docx]

Table S2. Skeletal articulation stages for fossil Dugongidae and the more inclusive group of all fossil vertebrates at Al Maszhabiya.

| Taxon | Stage 1 | Stage 2 | Stage 3 | Totals |
| --- | --- | --- | --- | --- |
| Dugongidae | 0 | 42 | 130 | 172 |
| All vertebrates | 0 | 42 | 137 | 179 |
