# Supplementary material for: High abundance of Early Miocene sea cows from Qatar shows repeated evolution of seagrass ecosystem engineers in Eastern Tethys
Source: PeerJ. 2025 Dec 10;13:e20030. doi: 10.7717/peerj.20030 (PMC12701702; doi:10.7717/peerj.20030)
Supplement: Supplemental Information 10 [file peerj-13-20030-s010.docx]

Table S3. Bone abrasion stages for fossil Dugongidae and the more inclusive group of all fossil vertebrates at Al Maszhabiya.

| Taxon | Stage 0 | Stage 1 | Stage 2 | Total scores |
| --- | --- | --- | --- | --- |
| Dugongidae | 0 | 103 | 6 | 109 |
| All vertebrates | 1 | 109 | 6 | 116 |
