# Supplementary material for: High abundance of Early Miocene sea cows from Qatar shows repeated evolution of seagrass ecosystem engineers in Eastern Tethys
Source: PeerJ. 2025 Dec 10;13:e20030. doi: 10.7717/peerj.20030 (PMC12701702; doi:10.7717/peerj.20030)
Supplement: Supplemental Information 11 [file peerj-13-20030-s011.docx]

Table S4. Descriptive statistics on n = 108 localities with fossil dugongid ribs at Al Maszhabiya in Fig. 1 and Fig. S5A.

| Statistic | Value |
| --- | --- |
| Mean | 2.36 cm |
| Median | 2.34 cm |
| Standard Deviation (σ) | 0.69 cm |
| Variance | 0.47 cm^2^ |
| Minimum | 1.24 cm |
| Maximum | 4.19 cm |
| Skewness | 0.82 |
| Kurtosis | 3.45 |
