# Supplementary material for: High abundance of Early Miocene sea cows from Qatar shows repeated evolution of seagrass ecosystem engineers in Eastern Tethys
Source: PeerJ. 2025 Dec 10;13:e20030. doi: 10.7717/peerj.20030 (PMC12701702; doi:10.7717/peerj.20030)
Supplement: Supplemental Information 12 [file peerj-13-20030-s012.docx]

Table S5. Extant sirenian specimens sampled for rib measurements in Figure S5B.

| Species | Histogram ID | Specimen | Body length |
| --- | --- | --- | --- |
| *Trichechus manatus* | Manatee 1 | USNM VZ 571671 | 241 cm |
| *Trichechus manatus* | Manatee 2 | USNM VZ 571675 | 292 cm |
| *Trichechus manatus* | Manatee 3 | USNM VZ 551663 | 307 cm |
| *Dugong dugon* | Dugong | USNM VZ 550456 | 233 cm |
