# Supplementary material for: High abundance of Early Miocene sea cows from Qatar shows repeated evolution of seagrass ecosystem engineers in Eastern Tethys
Source: PeerJ. 2025 Dec 10;13:e20030. doi: 10.7717/peerj.20030 (PMC12701702; doi:10.7717/peerj.20030)
Supplement: Supplemental Information 13 — MNI, minimum number of individuals; MNE, minimum number of elements. [file peerj-13-20030-s013.docx]

Table S6. Abundances of fossil vertebrates by occurrences from field localities at Al Maszhabiya. MNI, minimum number of individuals; MNE, minimum number of elements.

| Taxon | MNI | MNE |
| --- | --- | --- |
| Cetacea | 1 | 1 |
| Testudines | 2 | 2 |
| Dugongidae | 6 | 304 |
| Elasmobranchii | 1 | 1 |
| Osteichthyes | 1 | 3 |
| Totals | 11 | 311 |
