# Supplementary material for: High abundance of Early Miocene sea cows from Qatar shows repeated evolution of seagrass ecosystem engineers in Eastern Tethys
Source: PeerJ. 2025 Dec 10;13:e20030. doi: 10.7717/peerj.20030 (PMC12701702; doi:10.7717/peerj.20030)
Supplement: Supplemental Information 14 — (FD 23-14, FD 23-56, F 23-75) using rose diagrams both without polarity and with polarity. [file peerj-13-20030-s014.docx]

Table S7. Descriptive statistics for bone orientation in three fossil dugongid localities at Al Maszhabiya (FD 23-14, FD 23-56, F 23-75) using rose diagrams both without polarity and with polarity.

| Statistic | No polarity | Polarity |
| --- | --- | --- |
| Mean Direction | 98.95° | 82.48° |
| Dominant Direction | 112.5° | 67.5° |
| Resultant Vector Length | 0.732 | 0.553 |
| Circular Variance | 0.268 | 0.447 |
| Median Direction | 112.5 ° | 67.5° |
