# Supplementary material for: High abundance of Early Miocene sea cows from Qatar shows repeated evolution of seagrass ecosystem engineers in Eastern Tethys
Source: PeerJ. 2025 Dec 10;13:e20030. doi: 10.7717/peerj.20030 (PMC12701702; doi:10.7717/peerj.20030)
Supplement: Supplemental Information 15 [file peerj-13-20030-s015.docx]

Table S8. Qatar Dugong Beach Surveys 2014-2017

| Region | Coast Length (km) | No. Carcasses | Percentage (%) | Density (carcasses/km) | Region Delineation |
| --- | --- | --- | --- | --- | --- |
| SW | 83 | 5 | 5.6 | 0.06 | Dukhan Beach to KSA border |
| NW | 215 | 74 | 82.2 | 0.34 | Al Ruwais to Dukhan Beach |
| NE | 204 | 7 | 7.8 | 0.03 | Al Ruwais to West Bay Lagoon - North Doha |
| SE | 66 | 4 | 4.4 | 0.06 | West Bay Lagoon - North Doha to KSA border |
| Total | 568 | 90 | 100 | 0.50 | SW border to SE border |
