# Supplementary material for: High abundance of Early Miocene sea cows from Qatar shows repeated evolution of seagrass ecosystem engineers in Eastern Tethys
Source: PeerJ. 2025 Dec 10;13:e20030. doi: 10.7717/peerj.20030 (PMC12701702; doi:10.7717/peerj.20030)
Supplement: Supplemental Information 16 [file peerj-13-20030-s016.docx]

Table S9. Qatar Dugong Strandings 2014-2017 comparing West versus East Coasts.

| Region | Coast Length (km) | No. Carcasses | Percentage (%) | Density (carcasses/km) | Coast Delineation |
| --- | --- | --- | --- | --- | --- |
| West Coast | 298 | 79 | 87.8 | 0.40 | KSA border to Al Ruwais |
| East Coast | 270 | 11 | 12.2 | 0.10 | Al Ruwais to KSA Border |
