# Supplementary material for: High abundance of Early Miocene sea cows from Qatar shows repeated evolution of seagrass ecosystem engineers in Eastern Tethys
Source: PeerJ. 2025 Dec 10;13:e20030. doi: 10.7717/peerj.20030 (PMC12701702; doi:10.7717/peerj.20030)
Supplement: Supplemental Information 17 — Values are maxima number of individual skeletons; see supplementary text for more details. Data modified from Pyenson et al. (2014); supplementary data p. 25, table S12). [file peerj-13-20030-s017.docx]

Table S10. Reported and communicated densities for fossil sirenian sites from Supplemental Information text. Values are maxima number of individual skeletons; see supplementary text for more details. Data modified from Pyenson et al. (2014; supplementary data p. 25, table S12).

| Site | Geologic age | MNI | Notes | Sample area (km2) |
| --- | --- | --- | --- | --- |
| Al Maszhabiya, Qatar | Aquitanian | 7 | Bonebed level of Lower Al Kharrara Member | 0.79 |
| Seven Ribs, Qatar | Aquitanian | 1 | Lower Al Kharrara Member | 1.1 |
| CS-41, Spain | Lutetian | 6 | Represents a deltaic overbank deposit | 0.000024 |
| Wadi Al-Hitan, Egypt | Priabonian | See Comparable fossil bonebeds section. | About 24 per 100 km^2^ or about 1 per 4 km^2^ | 200 |
