# Supplementary material for: High abundance of Early Miocene sea cows from Qatar shows repeated evolution of seagrass ecosystem engineers in Eastern Tethys
Source: PeerJ. 2025 Dec 10;13:e20030. doi: 10.7717/peerj.20030 (PMC12701702; doi:10.7717/peerj.20030)
Supplement: Supplemental Information 18 — Geographic occurences, geology, paleoecology and taphonomy, rib surveys, diversity and abundance, skeletal orientation, dugong strandings in Qatar, comparable bonebeds, systematics, phylogenetics, and high-resolution scanning. [file peerj-13-20030-s018.docx]

**Supplemental Text**

**High abundance of Early Miocene sea cows from Qatar shows repeated evolution of seagrass ecosystem engineers in Eastern Tethys**

Nicholas D. Pyenson^1^*, Ferhan Sakal^2^, Jacques LeBlanc^3^, Jon Blundell^4^, Katherine D. Klim^5^, Christopher D. Marshall^6,7^, Jorge Velez-Juarbe^8^, Katherine Wolfe^4^, Faisal Al Naimi^2^

^1^ Department of Paleobiology, National Museum of Natural History, Smithsonian Institution, Washington DC, USA

^2^ Department of Archaeology, Qatar Museums, Doha, State of Qatar

^3^ Unaffiliated, Republic of Panama

^4^ Digitization Program Office, Office of Digital Transformation, Smithsonian Institution, Washington DC, USA

^5^ Stone Ridge School of the Sacred Heart, Bethesda, Maryland, USA

^6^ Department of Marine Biology, Texas A&M University at Galveston, Galveston, Texas, USA

^7^ Department of Ecology and Conservation Biology, Texas A&M University, College Station, Texas, USA

^8^ Department of Mammalogy, Natural History Museum of Los Angeles County, Los Angeles, California, USA

Corresponding Author:

Nicholas D. Pyenson

10^th^ and Constitution Ave NW, NHB MRC 121, Washington, DC 20560, United States of America

Email address: pyensonn@si.edu

Geographic occurrence data of dugongids in the Gulf Region

Zooarchaeology of dugongs in the Gulf Region

Figure 1A from the main text plots occurrence data from Pyenson et al. (2022) in yellow diamonds showing the distribution of zooarchaeological sites with dugong material throughout the Gulf Region. Historically, dugongs have had a cultural and economic importance in the Gulf Region since the Neolithic period, approximately 7,500 years ago (Méry et al. 2009; Beech et al. 2010).

Cenozoic occurrences of dugongids in the Gulf Region

As Pyenson et al. (2022) indicated marine mammals in the Gulf Region mostly have a Holocene record. On the periphery of the Gulf, Abbassi et al. (2016) reported a dugongid skeleton from Burdigalian limestones of the Qom Formation near Shirin Su in north-central Iran. The Shirin Su material are natural molds of ribs, which have limited use for diagnosing taxonomic assignment beyond Dugongidae. In Qatar, work since the time of Cavelier (1970) has identified fossil-bearing nearshore marine deposits that have been correlated throughout the Gulf Region (Al-Saad & Ibrahim, 2002; Dill et al. 2003). These deposits provide abundant preservation potential for fossil marine mammals in the region, yet the published record is limited to a preliminary report by LeBlanc (2009). Later, LeBlanc (2021) indicated extensive fossil material throughout Qatar, including fossil marine vertebrates from Eocene to Miocene in age. In particular, LeBlanc (2009, 2021) noted abundant fossil Dugongidae from early Miocene (Aquitanian-Burdigalian) localities in the Dam Formation of southwest Qatar, including an initial survey of the Al Maszhabiya area in 2009 (see below, Field Data).

Geologic context

Stratigraphy

The fossil dugongid-rich outcrops of the Al Maszhabiya bonebed are restricted to a horizon in the Lower Al-Kharrara Member, which is one of two members that belong to the lower part of the Early Miocene Dam Formation in southwest Qatar. The Dam Formation was initially subdivided into a lower and upper part by Cavelier (1970), until Dill et al. (2005) and Dill and Henjes-Kunst (2007) revisited a succession of sedimentary rocks belonging to the Dam Formation in the Jebel Al-Nakhash area (about 30 km northeast of Al Maszhabiya along the Salwa Road) and subdivided the Dam Formation into seven lithofacies associations. These lithofacies associations were stratigraphically grouped by Dill et al. (2005) from bottom to top into three members called the Salwa, Al Nakhash, and Abu Samrah members. LeBlanc (2021) renamed Dill et al. (2005)'s Salwa Member as “Al-Kharrara” as previously recommended by Al-Saad & Ibrahim (2002) because the name Salwa was already used as a member of the subsurface Cretaceous Simsima Formation in Qatar. Also, the Al-Nakhash and Al-Kharrara members both allowed for a refinement of the stratigraphy as each is comprised of a lower, middle, and upper unit.

Associated fauna

We collected an assemblage of marine vertebrates associated with the Al Maszhabiya bonebed that includes toothed whales, turtles, bony fishes and sharks (Fig. S1). An odontocete cetacean of indeterminate taxonomic affinity is represented by a small ulna (estimated at 5.5 cm long) belonging to a skeletally mature individual (ARC.2024.28.022). The presence of Testudines is represented by a small section of carapace (ARC.2023.28.016) and a fragmentary plastron (ARC.2023.28.017), both of which are not sufficiently diagnostic to differentiate between Chelonidae or possibly Pleurodira (which is represented from at least one locality in the Upper Al-Kharrara Member in the Southern Reserve). We also collected postcranial elements of bony fishes (ARC.2023.28.013), teeth belonging to barracuda (Sphyraenidae) relatives (ARC.2023.28.015), and a single cf. Carcharhiniformes tooth (ARC.2023.28.012) from the type locality of *Salwasiren* (FD 23-56). Fossil barracuda teeth have been frequently assigned to the genus *Sphyraena* (Ballen 2019), but Ballen (2020) noted that this tooth morphology is observed in other bony fish families (e.g., Gempylidae and Trichiuridae).

In the lower Dam Formation section that contains the Lower Al-Kharrara Member (Figure 2), Cavelier (1970) documented an extensive fossil invertebrate assemblage including nearshore and reef-building taxa: *Anomia* sp., *Clementia papyracea*, pelecypods, gastropods, *Ostrea latimarginata*, *Echinocyamus* sp., and crabs. The invertebrate taxa identified from the bonebed in the Lower Al-Kharrara Member are consistent with invertebrate fossils identified from correlative siliciclastic sequences of the Dam Formation reported by Al-Saad & Ibrahim (2002), Dill et al. (2005), and Dill and Henjes-Kunst (2007) in the Jebel Al-Nakhash area. Dill et al. (2005: table 2) provided a list of invertebrate fossil taxa that built on Cavelier (1970)’s list, including *Cardita* sp., *Cardium* sp., *Xenophora* sp., *Conus* sp., and *Turitella* sp. Partial fragments of crab claws are abundant in the Al Maszhabiya bonebed. At locality FD 23-56 (the type locality for *Salwasiren*), we also documented extensive horizontal *Thalassinoides* burrows within 0-5 cm below the semi-articulated skeletal elements of the *Salwasiren* type specimen; Dill et al. (2005: table 2) also reported invertebrate trace fossils belonging to *Planolites* and *Thalassinoides* (see also Dill & Henjes-Kunst, 2007). We noted that some localities featured extensive bioencrustation on ribs; at the type locality for *Salwasiren*, abundant *Ostrea latimarginata* shells were distributed near the cranium and humerus (Fig. S2). In both cases, this bioencrustration suggests exposure of bone on the seafloor prior to burial ~1 yr (see Boessenecker, 2013). Overall, the fossil assemblage associated with the Al Maszhabiya bonebed represents taxa that range in water depths 10–100 m (Dill et al. 2005), which is consistent with a lithology of the fine-grained siliciclastics 0-5 m from the section at Al Maszhabiya area representing water depths 5-25 m.

Geologic age

Overall, the Lower Al-Kharrara Member is Aquitanian, based on benthic foraminifera biostratigraphy (i.e., *Borelis melo melo*; see Jones, Simmons & Whittaker, 2006) and its correlation with the top of Dill et al. (2005)’s Lower Salwa Member of the Dam Formation. By fitting a marine strontium isotope curve of ^87^Sr/^86^Sr ratios from celestite I in sediments collected from the Jebel Al-Nakhash area, Dill et al. (2005) calculated a 21.6 Ma age for the top of a dolomite sequence in the Salwa 1a cyclothem unit within their Lower Salwa (= Al-Kharrara) Member of the Dam Formation. LeBlanc (2021) directly correlated Salwa 1a and 1b with Lower Al-Kharrara Member of the Dam Formation with the siliciclastics from Jebel Al-Nakhash restricted to the Salwa 1a cyclothem, overlain by calcite and dolomite deposits in Salwa 1b.We argue that this same boundary applies to Lower Al-Kharrara Member at the Al Maszhabiya area because similar siliciclastics at the bonebed horizon are overlain by calcite and dolomite packstones, and thus the Al Maszhabiya bonebed is no younger than the 21.6 Ma date calculated for the boundary between Salwa 1a and 1b at the Jebel Al-Nakhash area. Moreover, the Al Maszhabiya bonebed must be older than 21 Ma as that date constrains the top of Dill et al. (2005)'s Middle Salwa (= Al-Kharrara) Member. It is possible that the bonebed’s age is closer to 22.2 Ma (near the Aq2 global sea-level fall). The base of the Dam Formation is not older than the Chattian-Aquitanian boundary of 23.03 Ma. Like elsewhere in southwest Qatar, the Dam Formation unconformably rests on the Eocene Dammam Formation, which is a sedimentary unit that forms most of the peninsula of Qatar and extends over large portions of neighboring Saudi Arabia and Bahrain. Thus, we constrain the age of the Al Maszhabiya bonebed at 23.03-21.6 Ma.

Paleogeography

By the Aquitanian, the open Tethys Seaway became impaired by the emersion of the Arabian Shelf following the separation of Arabia from Africa earlier in the Cenozoic and the collision of Afro-Arabia with the Eurasia plate (Straume et al. 2025). The latter collision was protracted throughout the Paleogene, with the Eastern Tethys oceanic lithosphere subducted under Eurasia by the middle Oligocene (~28-26 Ma), placing the Arabian passive margin (along with the abducted Late Cretaceous ophiolites in Oman) in the foreland position (Fig. 5; see review in Straume et al. 2025). From the Oligocene through early Miocene (Aquitanian-Burdigalian), the closure of the oceanic part of the Tethyan Seaway formed an intermediate-shallow seaway sometimes called the Mesopotamian Trough (or Sea) that connected the Proto-Mediterranean Sea with the Indian Ocean. The Mesopotamian Trough was a shallow platform that provided intermittent marine connections between the Western and Eastern Tethys until about 19 Ma with the emergence of the *Gomphotherium* landbridge (Rögl, 1999; Harzhauser et al. 2007). This landbridge permitted the exchange of Afro-Arabian terrestrial species (especially large mammals) with Eurasia throughout the rest of the Neogene except for a short-lived seaway connection during eustatic sea-level highs of the Middle Miocene.

Prior to the closure of the Tethys Seaway over Arabia at 19 Ma, ocean circulation models suggest a westward surface flow from the Indian Ocean towards the Mediterranean Sea and Atlantic Ocean, but with intermediate and deep saline water flowing eastwards, towards the Indian Ocean, creating a warm and saline water mass in that ocean. The closure of the Tethys Seaway would have freshened and cooled the Indian Ocean, potentially intensifying the Antarctic Circumpolar Current (see references in Straume et al., 2025). Climate modelling by Sarr et al. (2022) indicated that the closure of the Mesopotamian Trough in the Burdigalian would have triggered marine upwelling along the Arabian coast, enhancing marine productivity and initiating large-scale oceanic circulation in the Indian Ocean that persists today. Harzhauser et al. (2007) noted that the biogeographic separation of the Western and Eastern Tethys marine invertebrate faunas was already underway in the Oligocene, prior to the emergence of the *Gomphotherium* landbridge, especially with tridacnine and strombid molluscs. By the Burdigalian, after the closure of the Mesopotamian Trough, the Proto-Mediterranean faunas had very few commonalities with those of the Indian and Pacific Oceans. Renema et al. (2008) argued that the Oligocene to Early Miocene fossil assemblages of Afro-Arabia, Iran, Oman, and Pakistan represented the second phase of a three-phase marine biodiversity hotspot that migrated from the basins in Western Europe to the Indo-Australian Archipelago today. The high density of Aquitanian fossil dugongids from the Al Maszhabiya bonebed represents additional abundance data for this marine biodiversity hotspot, along with associated marine vertebrate and invertebrate taxa (above; Fig. 5).

Field data

The densest area of fossil dugongid-bearing outcrops of the Lower Al-Kharrara Member of the Dam Formation is the Al Maszhabiya area, which is 0.76 km^2^ bounded by a vehicle exclusion zone near the coordinates of 24°45'36.3"N, 50°54'36.2"E in Al Rayyan Municipality of the State of Qatar (Figure 1). The high abundance of fossil dugongids at Al Maszhabiya was also observed by one of the co-authors (J. LeBlanc) in September 2009, who documented 47 localities at the time. QM collected one (or more) specimens from the Al Maszhabiya bonebed to create a composite skeleton now on display in the National Museum of Qatar in Doha (Qatar Museums, unpublished report). By the end of our 2024 field season, we documented a total of 172 fossil-bearing localities in the Al Maszhabiya, which we harmonized and reconciled with JL localities from 2009. Global positioning system (GPS) coordinates of all localities can be requested from QM. Erosion in the intervening time prevented us from reidentifying all JL localities, but the digital images from that initial reconnaissance were nonetheless used in our analyses. Table S1 reports on the relative proportions of fossil dugongid skeletal elements in our total surveys. Figure S3 illustrates the variety of fossil elements captured in our surveys.

Based on our fieldwork, we identified the Lower Al-Kharrara Member south of the Al Maszhabiya area at outcrops near a local point called Hazm Maszhabiya Al Janoubi. Based on J. LeBlanc’s fieldwork mapping the extent of the Dam Formation in southwestern Qatar, we also identified the Lower Al-Kharrara Member northwards beyond the Southern Reserve to exposures north of the Salwa Road in an area called the Seven Ribs (Figure 1). In the areas of both north and south of Al Maszhabiya, outcrops of Lower Al-Kharrara Member have lower densities of dugongid skeletal remains.

Cavelier (1970)’s described a dense unit at the base of the lower part of the Dam Formation that contained “[reptiles] and shark teeth” from Section 1 ibis. We think it is likely that Cavelier (1970) mistook fossil dugongid skeletal material for being fossil reptiles, which are absent from the Lower Al-Kharrara Member but present in the Upper Al-Kharrara Member (J. LeBlanc, N. Pyenson and F. Sakal, 2025, unpublished data). Fossil crocodylians have been reported about 200 km westwards from estruarine and fluvial deposits of the Dam Formation in Saudi Arabia (Thomas, 1982). The observation of shark teeth suggests higher abundances than noted in the Al Maszhabiya area. Importantly, we note that Cavelier (1970) referred to two stratigraphic sections that measured over 70 m of upper and lower Dam Formation exposed on the eastern flank of Hazm Mishabiyah located “7.5 km E from Abu Samra.” This latter landmark is challenging to relocate among several topographic highs ~50 m, including one currently identified as Hazm Al Maszhabiya located in Al Eraiq Reserve and >25 km east of Abu Samra. Based on our fieldwork and mapping available The Centre for GIS of Qatar’s Ministry of Municipality (https://www.gisqatar.org.qa/en/), we argue that Cavelier (1970)’s Hazm Mishabiyah is currently identified as Hazm Maszhabiya Al Janoubi (95 m high) near the coordinates 24°44'17.0"N 50°53'43.3"E. Hazm Maszhabiya Al Janoubi is located about 8.8 km east southeast of Abu Samra with a tall eastern flank that exposes a sequence of the Dam Formation with outcrops of the Lower Al-Kharrara Member walkable to a fossil dugongid locality (FD 24-006) about 1.5 km east of the topographic high (N. Pyenson and F. Sakal, 2025, unpublished data).

Paleoecological and taphonomic analyses

Although cetaceans have figured prominently in taphonomic studies for decades, few studies have investigated taxonomically comprehensive actualistic taphonomy of other marine mammal groups, such as pinnipeds and sirenians in a quantitative or operational manner. Lacking longitudinal studies that document the decay and decomposition of marine mammal carcasses in nearshore environments, we applied taphonomic scoring schemes that previously been used for the taphonomic histories of cetaceans (e.g., Boessenecker, Perry & Schmitt, 2014).

Skeletal articulation

Skeletal articulation in vertebrate remains can indicate different taphonomic signatures attributable to specific postmortem decay pathways. These signatures can be complex, and they can reflect the control of different factors related to ontogeny, taxonomy, and habitat preference. The degree of skeletal articulation is fundamentally an estimated, qualitative score that can vary in scope and comparability across many different vertebrate clades. Several scoring schemes have been applied to cetaceans, with the implication that such taphonomic processes also operated on other marine vertebrates, such as sirenians.

Here we adopted a simple, three-stage categorization to capture the range of skeletal articulation modes exhibited by fossil dugongids and other fossil marine vertebrates from the Al Maszhabiya bonebed, similar to the one Pyenson et al. (2014) used at Cerro Ballena in Chile, which also comprised by either isolated, individual elements or associated, but disarticulated skeletons. We adopted minor modifications from Boessenecker, Perry & Schmitt (2014: figure 5b). Our scoring scheme covered three categories:

- Articulation Stage 1 – Articulated, either completely or mostly, including axial and appendicular articulations.
- Articulation Stage 2 – Disarticulated, but associated, where all the elements are articulated sections, or separated but clearly associated, and thus belonging the same individual.
- Articulation Stage 3 – Isolated, separated elements, with no implied association, although an association cannot be excluded.

Our results are reported in Table S2.

Bone modification

Taphonomic studies have employed various schemes to score bone modification to describe alterations to the bone surface and structure that are comparable across sites, time periods, and taxonomic groups. Such comparisons provide the basis to infer processes that generate these patterns and discriminate between different taphonomic histories.

Most taphonomic studies incorporating bone modification scoring have focused on terrestrial ecosystems, including both modern and fossil ones. Studies employing such schemes for marine vertebrates are in clear minority, although some examples (Pyenson et al., 2014; Boessenecker, Perry & Schmitt, 2014) have demonstrated the value of such approaches. For the Al Maszhabiya bonebed, our bone modification comparisons focused on categories that were clearly useful for discriminating taphonomic features of the skeletal material in the area, similar to those used at Cerro Ballena. In our survey of >300 skeletal elements, we did not find any evidence of bite marks nor any evidence of scavenging, but we observed repeated evidence of bioencrustation, indicating multiple months to a year of exposure on the seafloor prior to burial (see above Geologic context: Associated fauna).

We scored bone abrasion for all fossil dugongids and fossil marine vertebrates from the Al Maszhabiya bonebed. We followed Fiorillo (1988:74-75) for fossil bone abrasion stages, paraphrased below:

- Abrasion Stage 0 – Angular, fresh bone, unabridged. All edges are defined.
- Abrasion Stage 1 – Bone edges are subangular, showing slight abrasion. Some edges are rounded and polished.
- Abrasion Stage 2 – Bone edges are subrounded, showing moderate abrasion. Osteological processes are recognizable but not in their original state. Some bone surfaces may be polished.
- Abrasion Stage 3 – Bone edges are rounded, with sufficient removal of the surface to reveal mostly trabecular bone. Remnant may be a bone pebble.

For these data, we explicitly transposed abrasion scores built for mammalian bone in terrestrial environments to marine ones. Because the original stages proposed (Fiorillo, 1988) were based on taphonomic studies of modern mammal bone weathering in terrestrial environments, it is unclear whether taphonomic processes operating on bone in marine environments produce the same weathering patterns. Thus, we did not examine bone weathering for material from the Al Maszhabiya bonebed, following similar reasoning for other fossil marine vertebrate bonebeds (Pyenson et al., 2014; Boessenecker, Perry & Schmitt, 2014).

Our results are reported in Table S3.

Survey of fossil dugongid ribs and modern comparisons

Measuring fossil dugongid ribs

Fossil ribs dominate the assemblage at the Early Miocene fossil site Al Maszhabiya. To better understand the distribution and parameters of this fossil assemblage, we developed a profile of fossil sea cow rib bone size across Al Maszhabiya fossil localities to reveal the frequency and modes of fossil dugongid rib sizes. Different sizes would likely reflect either a sample from a living population across a range of body sizes or different taxa living sympatrically.

In anterior or posterior view, sirenian ribs generally have a profile like a bass clef symbol (i.e., unevenly C-shaped). The angle of the rib is generally the inflection point on this asymmetry. The angle is also one of the places where sirenian ribs reach their maximum dimensions. Using a database of calibrated digital images collected for each fossil dugongid locality at Al Maszhabiya (i.e., FD 23-number series), we measured each image in Image J and calibrated the scale with the scale bar in the image. We then measured the largest dimension (i.e., most exposed skeletal surface) of each rib in the image, ideally near the angle of the rib, and entered the value to 2 decimal places. For localities with multiple ribs, we averaged the rib size. Note that some fossil localities are harmonized across FD 23 and JL locality naming systems; locality synonymies were noted. The resultant spreadsheet of fossil dugongid rib measurements by locality number is available on Zenodo (DOI: 10.5281/zenodo.15312915).

Because fossil sirenian ribs tend to come to rest on their anterior or posterior surface (i.e., their shape determines their center of gravity), fossil sirenian ribs tend to be oriented on depositional surfaces in the same manner (i.e., anterior or posterior surface as stratigraphic up). When exposed in the sediment layer, their *in situ* orientation reveals their lateral thickness, which we measured in n = 108 fossil localities. We measured rib thickness following Pyenson et al (2022), who cited fig. 11 and table 9 in Zalmout & Gingerich (2012) for rib measurements including lateral and anteroposterior thicknesses at the rib at the angle (at the level of the neck diameter in 22), as well as the lateral and anteroposterior thicknesses of the rib at midshaft (equivalent to MAWM, maximum anteroposteriorly width of midshaft, and MLWM, maximum mediolateral width at midshaft in 22).

The distribution of these fossil rib size data ranged between 1.24-4.19 cm, which is comparable to the size range for a single extant species (Fig. S5; see below). The skew of the fossil data is moderately positive, with the mean just slightly larger than the median value. The distribution is also mesokurtic, showing that the fossil data are essentially normally distributed (Table S4).

Modern ribs

To compare our survey of fossil dugongid ribs at Al Maszhabiya with extant sirenians, we measured a sample of ribs from vouchers with known sizes. To mimic comparable measurements of maximum rib thickness in lateral dimensions that we collected from field localities at Al Maszhabiya (i.e., FD 23 series localities), we measured a complete (or near-complete) series of ribs from extant sirenians in the Division of Mammals collections of the Department of Vertebrate Zoology at the Smithsonian Institution’s National Museum of Natural History (USNM VZ).

We identified specimens across a range of body lengths, focusing on complete series of ribs; when the option for a right or left side was available, we selected the more complete series or the more accessible one (e.g., only the right side was accessible dugong for a mounted dugong skeleton in the collections). For unmounted specimens, we carefully placed each rib on a flat surface and measured the maximum lateral thickness in the same manner as with fossils (above). Where possible, these ribs were measured in anatomical sequence, although for the purposes of this comparison (via frequency in a histogram) identifying specific rib numbers was not necessary.

The resultant histogram (Fig. S5) showed the size distribution of ribs for manatees alone (USNM VZ 571671, 571675, 551663; Table S5) covered the complete rib size range displayed at Al Maszhabiya. We were only able to measure the rib size distribution for one dugong specimen (USNM VZ 550456), which we estimate to have been 13 years old, based on comparable size data outlined by Sarko et al. (2010: table 1). Overall, the dugong rib size data fell within the distribution for the range of manatee size data, which was effectively the same range as fossil data from the Al Maszhabiya bonebed (Fig. S5).

Measuring abundance and diversity data

In palaeoecological studies measuring relative abundance at fossil sites where individual skeletons can be easily discerned, the minimum number of individuals (MNI) is a more informative metric than the minimum number of elements (MNE, see 24), especially for discerning taphonomic history. In assemblages where individual marine vertebrates are represented by intact vertebral columns (e.g., Pyenson et al. 2014; Kelley et al. 2022), skeletal associations plotted on the quarry map charts can be delineated. To measure taxonomic abundance Al Maszhabiya, we collected both MNI and MNE data for specific taxonomic groups, including Dugongidae, Cetacea, Testudines, Osteichthyes and Carchariniformes (see Table S6). To generate MNI, we tabulated the number of individual separate crania (n = 7), which we judged to be the most conservative count for MNI. It could be argued that each separate locality that we identified (i.e., discrete skeletal clusters <5 m apart; see for example Fig. S3 *A*) represented a single individual based on the dense association of skeletal elements <3 m^2^ for the type specimen of *Salwasiren* at FD 23-56, which is consistent with modern examples (see Fig. S2 of dugong skeletal elements on the Hawar Islands of Bahrain). However, it is not clear that this discrete mode of skeletal clustering is unique or even the dominant taphonomic mode, especially given the potential dispersal of skeletal elements by ocean currents at the time (see above). We decided to report the most conservative value for MNI, while arguing that the actual value may be closer to n = 10^2^. For MNE, we summed the total number of individual skeletal elements represented across all localities in the area.

Skeletal orientation at Al Maszhabiya

We measured the orientation of the long axis of each major element in three localities (i.e., FD 23-14, FD 23-56, and FD 23-75) with associated fossil dugongid skeletons, relative to magnetic north. Similar to the rorqual skeletons at Cerro Ballena that had crania in articulation or nearly articulated with the postcranial skeleton (Pyenson et al. 2014), we measured the orientations of individual bones relative to this anterior direction for FD 23-14 (based on the rib orientations and position of the thoracic cavity relative to an ilium), FD 23-56 (based on the location of the cranium and its orientation), and FD 23-75, where we assumed an anterior direction for north based on the proximal ends of 4 ribs in close articulation (see Fig. S4 *B*). We also analyzed the orientations assuming no polarity (see Lindsey, 2015), only a declination from north. We then used the *circular* package in R (Agostinelli & Lund, 2024) to calculate the statistical distribution of the data reported in Table S7.

Overall, the distribution of fossil dugongid skeletons across three localities showed a clear dominant direction in a northeast orientation, with little scatter based on low vector length and circular variance values (Fig. S6). The dominant direction likely reflects a paleocurrent from the northeast, which is consistent with the expected westwards surface current from the Indian Ocean in the Mesopotamian Trough during the Aquitanian, prior to the *Gomphotherium* landbridge emergence (see above in Paleogeography and Figure 5). It is unlikely that such a current originated from the southwest direction, as that direction was emergent and part of Afro-Arabia during the Early Miocene.

Modern dugong beach strandings in Qatar

One of us (C. Marshall) initiated a modern dugong (*Dugong dugong*) stranding program in 2014 to assess the number of stranded dugongs and their location in Qatar. Beach surveys for stranded dugongs were conducted from 2014 to 2017 with an emphasis on the northwest and west coast. Additionally, members of Qatar University responded to calls from the public and government officials reporting stranded dugongs along any coastal zone in Qatar. Dugong remains included pieces of skeleton, whole skeletons, and whole bodies from fresh to mummified. There is clear evidence that bycatch is an issue as many stranded dugongs had evidence of negative exposure to fishery gear including entanglement in large nets, lines and ropes.

The entire coastline of Qatar is ~568 km long. For the purposes of identifying hotspots of dugong stranding, the country was divided into four quadrants and the number of strandings assigned to each quadrant to measure the density of carcasses (Table S8). The northwest quadrant of Qatar (from Al Ruwais in the north to Dukhan Beach on the west coast) had the highest percentage of strandings (82.2%) and the highest density of carcasses (0.34, n = 74), followed by the northeast (7.8%), southwest (5.6%), and southeast (4.4%). The west coast had 87.8% of all dugong strandings (Table S9). The region between Qatar and Bahrain has one of the world’s last remaining contiguous seagrass meadow, and it is in this region that large groups of dugongs gather in the largest single dugong herd recorded (28-31). Sex and size were not always able to be collected but the dataset includes known males and females, and all age classes including pregnant females and presumptive females with calves. The current distribution and density of both live and stranded dugongs is consistent with this paleobiology of sirenian use of this area of the Gulf.

Comparable fossil sirenian and fossil marine mammal bonebeds

We compared the density of fossil marine mammal skeletons preserved at Al Maszhabiya with those from productive and abundant fossil marine mammal localities elsewhere in the world. We excluded fossil sites that preserve condensed, time-averaged assemblages on hiatal surfaces. The Middle Miocene Sharktooth Hill bonebed, for example, likely preserves hundreds of thousands of individual fossil marine mammals, including cetaceans and pinnipeds, but the unit is a time-averaged deposit that accumulated during a depositional hiatus, encompasses 700 kyr, and preserves mostly disarticulated and disassociated skeletal remains (Pyenson et al. 2009). Other reported Mio-Pliocene localities with dense accumulations of marine vertebrate material, which likely represent attritional deposits on hiatal surfaces, such as the Bahía Inglesa Formation (localities southwest of Cerro Ballena, Pyenson et al. 2014).

We also focused specifically on dense fossil sirenian sites. For Paleogene sirenians, there are reported but unpublished bonebeds from the Eocene of Jamaica (Seven Rivers Formation, Donovan et al. 1990) and the Eocene of France (Les Sireniens, Castellane, France; Floquet & Lamotte, 2007); we expand below on two published reports from the Eocene of Spain and the Eocene of Egypt. For Neogene sirenian sites, there is a reported deposit from the Middle Miocene of Mexico (Domning, 1978; J. Velez-Juarbe, 2025, personal observations), but it has yet to be studied exhaustively. Our results are reported in Table S10.

Wadi Al-Hitan

Currently a UNESCO World Heritage Site, Wadi Al-Hitan of the Egyptian desert has produced a rich diversity of marine mammals, including early cetaceans (mostly basilosaurids) and early sirenians. Peters et al. (2009) reported "1,400 complete or partial vertebrate skeletons from an area of ~200 km^2^.” Gingerich (1992) and Peters et al. (2009) indicated that the highest density of marine mammal skeletons originates from the Birket Qarun Formation, near or below the Pr-2 SB of Priabonian stage. Peters et al. (2009) stated there were 20.6 vertebrate sites per km (along strike) at Pr-2 SB.

More specifically, Zalmout and Gingerich (2012) identified 38 total sirenian specimens mapped across the late Eocene outcrops in the area: 14 specimens from localities in Fayum, north of Lake Birket Qarun, and 24 specimens from Wadi Al-Hitan. The former 14 specimens from north of Lake Birket Qarun are a small and unrepresentative sample from a large area, so they are likely unreliable for an estimate of the density (P. Gingerich, personal correspondence with N. Pyenson, 2025). Assuming the reported area (200 km^2^) roughly includes all of the late Eocene outcrop in Wadi Al-Hitan and assuming rocks of Priabonian age Gehannam and Birket Qarun formations that yield fossils sirenians cover about half of this area, the resulting in a density of early sirenians is about 24 per 100 km^2^ or about 1 per 4 km^2^.

Castejón de Sobrarbe-41

From the Late Eocene (Lutetian) of Spain, Castejón de Sobrarbe-41 (CS-41) is a fossil-bearing level from the uppermost part of the Sobrarbe Formation that is middle Lutetian (or middle Eocene). Díaz-Berenguer et al. (2018) reported >640 vertebrate bone elements from a quarry of 24 m^2^ including at least six sirenian individuals represented by 300 disarticulated skeletal elements from different ontogenetic stages referred to *Sobrarbesiren.* Aside from turtle plates and bones, and scarce eusuchian crocodylomorph teeth, no other large vertebrates were collected from CS-41. Díaz-Berenguer et al. (2018) interpreted this assemblage as an overbank deposit in an intertidal channel during a single energetic event, where all vertebrate remains dispersed over the tidal flat were trapped.

First, the Al Maszhabiya area belongs to the Lower Al-Kharrara Member of the Dam Formation as the Seven Ribs area (but perhaps not the same horizon), which is north of the Salwa Road and about 10 km NW of the Al Maszhabiya. Notably, there are 15 fossil-bearing localities in this area, but MNI = 1. Thus, the fossil density from Al Maszhabiya is quantifiably greater. Also, the Al Maszhabiya bonebed has a similar magnitude of MNI and skeletal elements (>300) as CS-41 from Spain, while being distributed over a wider geographic area (about 30 km^2^); CS-41 is a deposit that likely reflects both gregarious social behavior and physical factors concentrating skeletal density. By contrast, the fossil sirenians from Wadi Al-Hitan are distributed over the largest area (about 100 km^2^), but they have a lower density than sites in both Qatar and Spain.

Systematics, phylogenetics, and high-resolution scanning of *Salwasiren qatarensis*

Museum abbreviations

ECOCHM, Museo de Zoología, El Colegio de la Frontera Sur (ECOSUR), Chetumal, Quintana Roo, Mexico; LACM, Natural History Museum of Los Angeles County, Los Angeles, California, U. S. A.; MHNBx, Muséum d’Histoire naturelle de Bordeaux, Bordeaux, France; MHN-UABCS-Sils, Museo de Historia Natural de la Universidad Autónoma de Baja California Sur, La Paz, Baja California Sur, Mexico; MNHN, Muséum National d'Histoire Naturelle, Paris, France; SC, South Carolina State Museum, Columbia, South Carolina, U.S.A.; UF/FGS, former Florida Geological Survey collection, now housed at the Florida Museum of Natural History, University of Florida, Gainesville, Florida, U.S.A.

Phylogenetic analysis

We used the character-state matrix from Suarez et al. (2021), which was derived from Velez-Juarbe and Wood (2018), which included an undescribed specimen of Dioplotherium (ECOCHM 2491), that was subsequently removed for our analysis. The matrix from Suarez et al. (2021) was further modified by adding the stem sirenians *Ashokia antiqua*, *Protosiren fraasi*, *Libysiren sickenbergi*, *Eotheroides clavigerum*, *E. sandersi*, *E. lambondrano* *Prototherium veronense*, *Eosiren imenti* Domning, and the dugongids *Metaxytherium crataegense*, *M. medium*, *M. subapenninum*, *Dusisiren d*ewana, *Stegosiren macei*, *Norosiren zazavavindrano*, *Dioplotherium* cf. *D. allisoni* and *Salwasiren qatarensis*, for a total of 64 ingroup taxa.

Our outgroups include the stem proboscidean *Phosphatherium escuilliei*, and the desmostylian *Cornwallius sookensis* as done in previous analyses (see above). Furthermore, we added and modified character 32 from Domning (1994), and added characters 223-228 from Díaz-Berenguer et al. (2018). In addition the following characters were updated: ch. 73, 74, 221-225 for *Prototherium intermedium* based on the figures and description of *Pr. solei* (j. syn. of *Pr. intermedium*; Balaguer & Alba, 2016); ch. 224-228 for *Dusisiren reinharti*, based on MHN-UABCS-Sils 01/01/01; ch. 115 and 225 for *D. dewana*, based on LACM 23847; ch. 227-228 for *Metaxytherium arctodites*, based on LACM 127720; ch. 226 and 228 for *Crenatosiren olseni* based on UF/FGS 6094 and SC 90.104; and ch. 10, 11, 22, 23, 44, 64, 66, 67, 72, 88, 99, 101, 102, 105, 115 for *Rytiodus capgrandi* based on MNHN.F.MBA 1-3 and MHNBx 2006.PG.208; and, ch. 227-228 for *Trichechus inunguis* based on LACM 30818.

We performed the analysis in TNT v.1.5 (Goloboff, Farris & Nixon, 2008). All characters were initially treated as unordered and analyzed under equal and implied weights (k = 3 and k =9) (Goloboff et al. 2008, Goloboff et al. 2018). We then performed a heuristic search of 10000 replicates using the tree bisection-reconnection (TBR) algorithm with a backbone constraint tree based on the molecular phylogeny from Springer et al. (2015). Bootstrap values were obtained by performing 10000 replicates (Fig. S7).

Supplemental References

Abbassi N, Domning DP, Izad NN, Shakeri S. 2016. Sirenia fossils from Qom formation (Burdigalian) of the Kabudar Ahang Area, Northwest Iran. *Riv. Ital. Paleontol. Stratigr.* 122:13–24.

Agostinelli C, Lund U. 2024. R package ‘circular’: Circular Statistics (version 0.5-1). <https://CRAN.R-project.org/package=circular>

Al-Saad H, Ibrahim MI. 2002. Stratigraphy, micropaleontology, and paleoecology of the Miocene Dam Formation, Qatar. *GeoArabia* 7:9–28.

Balaguer J, Alba DM. 2016. A new dugong species (Sirenia, Dugongidae) from the Eocene of Catalonia (NE Iberian Peninsula). *C. R. Palevol* 15:489–500.

Beech MJ. 2010. Mermaids of the Arabian Gulf: archaeological evidence for the exploitation of dugongs from prehistory to the present. *Liwa J. Natl. Cent. Doc. Res.* 2:3–18.

Ballen GA. 2019. Nomenclature of the Sphyraenidae (Teleostei: Carangaria): A synthesis of fossil- and extant-based classification systems. *Zootaxa* 4686:397–408.

Ballen GA. 2020. New records of the genus *Sphyraena* (Teleostei: Sphyraenidae) from the Caribbean with comments on dental characters in the genus. *Journal of Vertebrate Paleontology* 40:e1849246.

Boessenecker RW. 2013. Taphonomic implications of barnacle encrusted sea lion bones from the middle Pleistocene Port Orford Formation, coastal Oregon. *J. Paleontol.* 87:657–663.

Boessenecker RW, Perry FA, Schmitt JG. 2014. Comparative taphonomy, taphofacies, and bonebeds of the Mio-Pliocene Purisima Formation, Central California: strong physical control on marine vertebrate preservation in shallow marine settings. *PLoS One* 9:e91419.

Cavelier C. 1970. Geologic description of the Qatar Peninsula (Arabian Gulf). Publication of the Government of Qatar, Department of Petroleum Affairs 39.

Díaz-Berenguer E, et al. 2018. First adequately-known quadrupedal sirenian from Eurasia (Eocene, Bay of Biscay, Huesca, northeastern Spain). *Sci. Rep.* 8:5127.

Dill HG, Botz R, Berner Z, Stüben D, Nasir S, Al-Saad H. 2005. Sedimentary facies, mineralogy, and geochemistry of the sulphate-bearing Miocene Dam Formation in Qatar. *Sediment. Geol.* 174:63–96.

Dill HG, Henjes-Kunst F. 2007. Strontium (^87^Sr/^86^Sr) and calcium isotope ratios (^44^Ca/^40^Ca-^44^Ca/^42^Ca) of the Miocene Dam Formation in Qatar: tools for stratigraphic correlation and environment analysis. *GeoArabia* 12:61–76.

Dill HG, Nasir S, Al-Saad H. 2003. Lithological and structural evolution of the northern sector of Dukhan anticline, Qatar, during the early Tertiary: With special reference to sequence stratigraphic bounding surfaces. *GeoArabia* 8:201–226.

Domning DP. 1978. Sirenian evolution in the North Pacific Ocean. *Univ. Calif. Publ. Geol. Sci.* 118:1–176.

Domning DP. 1988. Fossil Sirenia of the west Atlantic and Caribbean region. I. *Metaxytherium floridanum* Hay, 1922. *J. Vertebr. Paleontol.* 8:395–426.

Domning DP. 1994. A phylogenetic analysis of the Sirenia. *Proc. San Diego Soc. Nat. Hist.* 29:177–189.

Donovan SK, Domning DP, Garcia FA, Dixon HL. 1990. A bone bed in the Eocene of Jamaica. *J. Paleontol.* 64:660–662.

Fiorillo AR. 1988. Taphonomy of Hazard Homestead Quarry (Ogallala Group), Hitchcock County, Nebraska. *Rocky Mt. Geol.* 26:57–97.

Floquet M, Lamotte D. 2007. Le gisement fossilifère à dugongs (siréniens) du synclinal de Taulanne: hécatombes, tsunamites/tempestites, enfouissement. *Livret-Guide Excursion Géologique* 3:57–83.

Gingerich PD. 1992. Marine mammals (Cetacea and Sirenia) from the Eocene of Gebel Mokattam and Fayum, Egypt: stratigraphy, age, and paleoenvironments. University of Michigan Papers in Paleontology 30:1-84.

Goloboff PA, Farris JS, Nixon KC. 2008. TNT, a free program for phylogenetic analysis. *Cladistics* 24:774–786.

Goloboff PA, Carpenter JM, Arias JS, Esquivel DR. 2008. Weighting against homoplasy improves phylogenetic analysis of morphological data sets. *Cladistics* 24:758–773.

Goloboff PA, Torres A, Arias JS. 2018. Weighted parsimony outperforms other methods of phylogenetic inference under models appropriate for morphology. *Cladistics* 34:407–437.

Harzhauser M, Kroh A, Mandic O, Piller WE, Göhlich U, Reuter M, Berning B. 2007. Biogeographic responses to geodynamics: a key study all around the Oligo–Miocene Tethyan Seaway. *Zool. Anz.* 246:241–256.

Jones RW, Simmons MD, Whittaker JE. 2006. On the stratigraphical and palaeobiogeographical significance of *Borelis melo melo* and *B. melo curdica* (Foraminifera). *J. Micropalaeontol.* 25:175–185.

Kelley NP, Irmis RB, De Polo PE, Noble PJ, Montague-Judd D, Little H, Blundell J, Rasmussen C, Percival LME, Mather TA, Pyenson ND. 2022. Grouping behavior in a Triassic marine apex predator. *Current Biology* 32:5398–5405

Khamis A, Abdulla A, D’Souza E, Kelkar N, Arthur R, Al Khalifa E, Bader H, Alcoverro T. 2023. Long-term persistence of large dugong groups in a conservation hotspot around Hawar Island, Kingdom of Bahrain. Aquatic Conservation: Marine and Freshwater Ecosystems 33:592-605.

LeBlanc J. 2009. A fossil hunting guide to the Miocene of Qatar, Middle East: a geological & macro-paleontological investigation of the Dam Formation.

LeBlanc J. 2021. Stratigraphic Lexicon: a revised guide to the cenozoic surface formations of Qatar, Middle East (excluding the islands). *Biosis: Biol. Syst.* 2:361–407.

Lindsey EL. 2015. Tanque Loma, a new late-Pleistocene megafaunal tar seep locality from southwest Ecuador. *J. South Am. Earth Sci.* 57:61–82.

Lyman RL. 1994. Relative Abundances of Skeletal Specimens and Taphonomic Analysis of Vertebrate Remains. *PALAIOS* 9:288–298.

Marshall CD, Al Ansi M, Dupont J, Warren C, Al Shaikh I, Cullen J. 2018. Large dugong (*Dugong dugon*) aggregations persist in coastal Qatar. *Mar. Mamm. Sci.* 34.

Méry S, Charpentier V, Auxiette G, Pelle E. 2009. A dugong bone mound: the Neolithic ritual site on Akab in Umm al-Quwain, United Arab Emirates. *Antiquity* 83:696–708.

Peters SE, Antar MSM, Zalmout IS, Gingerich PD. 2009. Sequence stratigraphic control on preservation of late Eocene whales and other vertebrates at Wadi Al-Hitan, Egypt. *PALAIOS* 24:290–302.

Preen A. 1989. Observations of mating behavior in dugongs (*Dugong dugon*). *Mar. Mamm. Sci.* 5:382–387.

Preen A. 2004. Distribution, abundance and conservation status of dugongs and dolphins in the southern and western Arabian Gulf. *Biol. Conserv.* 118:205–218.

Pyenson ND, Al-Ansi M, Fieseler CM, Al Jaber KH, Klim KD, LeBlanc J, Mohamed AMD, Al-Shaikh I, Marshall CD. 2022. Fossil Sirenia from the Pleistocene of Qatar: new questions about the antiquity of sea cows in the Gulf Region. *PeerJ* 10:e14075.

Pyenson ND, Irmis RB, Lipps JH, Barnes LG, Mitchell Jr ED, McLeod SA. 2009. Origin of a widespread marine bonebed deposited during the middle Miocene Climatic Optimum. *Geology* 37:519–522

Pyenson ND, Gutstein CS, Parham JF, Le Roux JP, Chavarría CC, Little H, Metallo A, Rossi V, Valenzuela-Toro AM, Vélez-Juarbe J, Santelli CM, Rogers DR, Cozzuol MA, Suárez ME. 2014. Repeated mass strandings of Miocene marine mammals from Atacama Region of Chile point to sudden death at sea. *Proceedings of the Royal Society B: Biological Sciences* 281:20133316

Renema W, Bellwood DR, Braga JC, Bromfield K, Hall R, Johnson KG, Lunt P, Meyer CP, McMonagle LB, Morley RJ, O’Dea A, Todd JA, Wesselingh FP, Wilson MEJ, Pandolfi JM. 2008. Hopping hotspots: global shifts in marine biodiversity. *Science* 321:654–657

Rögl F. 1999. Mediterranean and Paratethys: facts and hypotheses of an Oligocene to Miocene paleogeography: short overview. *Geol. Carpath.* 50:339–349.

Sarko DK, Domning DP, Marino L, Reep RL. 2010. Estimating body size of fossil sirenians. *Mar. Mamm. Sci.* 26:937–959.

Sarr AC, Donnadieu Y, Bolton CT, Ladant JB, Licht A, Fluteau F, Laugié M, Tardif D, Dupont-Nivet G. 2022. Neogene South Asian monsoon rainfall and wind histories diverged due to topographic effects*. Nature Geoscience* 15:314–319.

Springer MS, Signore AV, Paijmans JLA, Vélez-Juarbe J, Domning DP, Bauer CE, He K, Crerar L, Campos PF, Murphy WJ, Meredith RW, Gatesy J, Willerslev E, MacPhee RDE, Hofreiter M, Campbell KL. 2015. Interordinal gene capture, the phylogenetic position of Steller’s sea cow based on molecular and morphological data, and the macroevolutionary history of Sirenia. *Molecular Phylogenetics and Evolution* 91:178–193

Straume EO, Faccenna C, Becker TW, Steinberger B, Licht A, Sembroni A, Gvirtzman Z, Ballato P. 2025. Collision, mantle convection and Tethyan closure in the Eastern Mediterranean. Nature Reviews Earth & Environment 6:299–317. https://doi.org/10.1038/s43017-025-00653-2

Suárez C, Gelfo JN, Moreno-Bernal JW, Vélez-Juarbe J. 2021. An early Miocene manatee (Sirenia, Trichechidae) from the Urumaco sequence (Venezuela, South America). *Journal of South American Earth Sciences* 109:103277

Thomas H, Sen S, Khan M, Battail B, Ligabue G. 1982. The lower Miocene fauna of As-Sarrar (Eastern Province, Saudi Arabia). *Atlal: Journal of Saudi Arabian Archaeology* 5:109–136.

Vélez-Juarbe J, Wood AR. 2018. An early Miocene dugongine (Sirenia: Dugongidae) from Panama. *J. Vert. Paleontol.* 38:e1511799.

Zalmout IS, Gingerich PD. 2012. Late Eocene sea cows (Mammalia, Sirenia) from Wadi al Hitan in the western desert of Fayum, Egypt. University of Michigan Papers in Paleontology 37:1-158.
